# Part 2—Cardiac Rehabilitation After an Acute Myocardial Infarction: Timing and Gender Differences in Adherence; Where Do We Stand?

**DOI:** 10.3390/jcm14041189

**Published:** 2025-02-11

**Authors:** Aneta Aleksova, Alessandra Lucia Fluca, Antonio Paolo Beltrami, Elena Dozio, Gianfranco Sinagra, Maria Marketou, Milijana Janjusevic

**Affiliations:** 1 Cardiothoracovascular Department, Azienda Sanitaria Universitaria Giuliano Isontina, 34100 Trieste, Italy; alessandralucia.fluca@units.it (A.L.F.); gianfranco.sinagra@asugi.sanita.fvg.it (G.S.); mjanjusevic@units.it (M.J.); 2 Department of Medical Surgical and Health Sciences, University of Trieste, 34125 Trieste, Italy; 3 Dipartimento di Area Medica (DAME), Istituto di Patologia Clinica, University of Udine, 33100 Udine, Italy; antonio.beltrami@uniud.it; 4 Department of Biomedical Sciences for Health, University of Milan, 20122 Milan, Italy; elena.dozio@unimi.it; 5 Cardiology Department Crete, School of Medicine, Heraklion University General Hospital, University of Crete, 700 13 Heraklion, Greece; maryemarke@yahoo.gr

**Keywords:** cardiac rehabilitation, myocardial infarction, illness perception, adherence, gender difference

## Abstract

Cardiac rehabilitation is a beneficial multidisciplinary interventional protocol that improves cardiovascular health and reduces mortality and morbidity rates in patients with cardiovascular diseases. Multiple studies have demonstrated that the implementation of such protocols in patients with acute myocardial infarction (MI) dramatically improved patients’ outcome. It is unfortunate that in practice, in spite of the advantages of cardiac rehabilitation, this approach is seldom employed. Indeed, only some guidance, such as American College of Cardiology and European Society of Cardiology guidelines, recommends cardiac rehabilitation in their protocols. In particular, the European guideline recommends its early implementation while the patient is still in hospital, whereas the American guideline suggests that it should be approximately three weeks after discharge. In Part 1 of this two-part comprehensive review, we provided a historical overview of cardiac rehabilitation, a detailed examination of each component of the cardiac rehabilitation programme, and its impact on cardiovascular health. In Part 2, the objective was to provide a comprehensive explanation of the optimal timing for the commencement of the cardiac rehabilitation programme, and to elucidate the factors that influence low engagement in such programmes, as well as the gender-based differences in adherence.

## 1. Introduction

Cardiac rehabilitation is a comprehensive, safe, and multidisciplinary patient-tailored intervention that has been demonstrated to reduce morbidity and mortality risk among patients with cardiovascular diseases (CVD) [1,2]. In the context of myocardial infarction (MI), a substantial body of evidence from numerous studies has demonstrated the positive impact of this programme on cardiovascular health outcomes [1,2,3]. It is regrettable that, in practice, despite the benefits of cardiac rehabilitation, this approach is rarely implemented [4].

In Part 2 of this two-part comprehensive review, the objective was to provide a detailed explanation of the optimal timing for the commencement of the cardiac rehabilitation programmes. We focused on the factors influencing low engagement in such programmes with a particular emphasis on the gender-based differences in adherence. In Part 1, an examination of the historical development of cardiac rehabilitation was undertaken, with particular attention paid to the studies that have shaped and advanced the understanding of this programme over the past century. Indeed, it was originally thought that any movement during the acute phase of MI could endanger the patient’s condition [5,6,7]. However, subsequent studies demonstrated that the gradual introduction of mobilisation leads to positive patient outcomes. This has led to improved current knowledge and a better understanding of the benefits of patient-tailored exercise programmes [8,9]. In addition, in Part 1 we explained all four phases of cardiac rehabilitation in detail.

## 2. Impact of Timing of Cardiac Rehabilitation Initiation on Outcome

The precise point at which a patient should commence cardiac rehabilitation still remains unclear. Two distinct perspectives exist regarding the optimal timing of cardiac rehabilitation. One perspective maintains that rehabilitation should commence with Phase I during the hospital stay, whereas the opposing view is that cardiac rehabilitation should begin with Phase II, with a delay.

In this context, it is worth mentioning that, in one study, it was observed that a longer waiting time negatively affected the enrolment of patients in cardiac rehabilitation. For every additional day of waiting time, the likelihood of enrolment decreased by 1% [10]. Moreover, a study by Johnson et al. (2014) indicated that the optimal timeframe for commencing cardiac rehabilitation is within the initial three weeks following a cardiac event. More precisely, in this study, patients who had experienced cardiac events or undergone cardiac surgery, and who had commenced cardiac rehabilitation at different time points, were compared according to the timing of their entry into the programme. The beneficial outcomes of cardiac rehabilitation were observed irrespective of the commencement of the programme. However, a comparison of the results between patient groups revealed that those who initiated cardiac rehabilitation after 30 days exhibited lower metabolic equivalent levels, exercise capacity, and weight improvements compared to patients who commenced the programme earlier. Furthermore, delayed enrolment was found to be directly related to poorer patient outcomes [11].

However, in a recent systematic review and meta-analysis, Zhang et al. (2024) [12] sought to ascertain which approach, inpatient or outpatient, yields superior outcomes in terms of enhancing cardiac function. This analysis represents perhaps the most comprehensive to date. More precisely, eight of the sixteen included studies commenced rehabilitation two days after PCI, whereas the remaining eight studies commenced rehabilitation one month after the procedure. The authors revealed no statistically significant difference in the improvement of cardiovascular rehabilitation outcomes evaluated (i.e., arrhythmias, angina pectoris, and coronary artery restenosis) according to the timing of the initiation of the cardiac rehabilitation programme. Since the severity and complexity of underlying clinical conditions after MI are highly heterogeneous, they require personalised rehabilitation strategies. Indeed, while some patients may benefit from an early mobilisation programme following PCI, for other patients, a later approach may be more appropriate. Similarly, there was no discernible difference in outcomes associated with the duration of the rehabilitation programmes. These findings demonstrate the feasibility of initiating cardiac rehabilitation with continued long-term adherence. Nevertheless, the complex nature of the underlying factors that contribute to CVD and the beneficial pathways triggered by cardiac rehabilitation necessitate further clinical investigation in order to identify the optimal initiation time and duration of cardiac rehabilitation [12].

Finally, despite the evidence from numerous studies demonstrating the efficacy of cardiac rehabilitation, commencing with Phase I, in improving patients’ conditions after PCI for acute MI, and the safety of the strategy with minimal haemodynamic and respiratory complications [7,12,13], the majority of cardiovascular societies’ guidelines still do not recommend early mobilisation after acute MI [7]. The European Society of Cardiology Guidelines advocate for the initiation of cardiac rehabilitation in patients with acute MI as early as possible [1,2], recognising that this approach has a more pronounced impact on medical therapy and adherence at follow-up, with a significant improvement in prognosis, as well as a reduction in hospital stay and cost [14]. On the other hand, the American College of Cardiology Guidelines also recommend that all patients with cardiovascular diseases should be offered cardiac rehabilitation programmes, ideally commencing within one to two weeks of the event. It is notable that other official guidance does not mention cardiac rehabilitation programmes in their protocols [2].

In conclusion, the timing of initiation of cardiac rehabilitation remains a topic of debate in clinical practice. Early in-hospital mobilisation (phase I) may stimulate physiological adaptations that are beneficial for recovery. However, the argument for delaying the initiation of cardiac rehabilitation (post-discharge phase II rehabilitation) arises from concerns about patient stability and readiness for rehabilitation. Indeed, as mentioned above, some patients may require more time to recover from acute cardiac events before engaging in structured exercise programmes. Given the complex nature of CVD and the multiple pathways activated by cardiac rehabilitation, further clinical studies are warranted to explore optimal timing and tailor rehabilitation strategies to individual patient needs. Understanding these physiological mechanisms will help to refine guidelines and improve patient outcomes in cardiac rehabilitation.

Finally, it is important to emphasise that cardiac rehabilitation should be tailored based on additional patient factors such as the presence of comorbidities, the extent of the MI, and whether it is the patient’s first or subsequent event. For example, patients with comorbidities such as diabetes mellitus, history of stroke, etc., may require modified rehabilitation programmes. Patients with a larger MI may require a more delayed and gradual approach, and those with a previous MI may require more challenging strategies. Further research is needed to establish specific guidelines that take these variables into account in cardiac rehabilitation protocols.

## 3. Low Adherence Reasons

While timing is crucial, adherence to cardiac rehabilitation programmes also significantly influences outcomes. Unfortunately, despite the evidence of the efficacy of cardiac rehabilitation, there is still some resistance to implementing this strategy, especially in Phase I. The reasons for poor adherence are likely to be multiple, and may include a lack of education or comprehension regarding the benefits of early mobilisation resulting in fear of pain, reluctance, and anxiety towards the idea of exercising upon acute MI, as well as low interest and motivation [4]. Nevertheless, the issue of patient consent for early cardiac rehabilitation is not the sole concern. Indeed, in the majority of cases, cardiac rehabilitation is not even offered by healthcare professionals in the early stages due to a lack of expertise, knowledge and interest, and finally due to the limited resources of the hospital and reimbursement from the health system. It is evident that there is a need for further education for both patients and practitioners in this area [4].

In addition, even in cases where a cardiac rehabilitation programme has been offered at an early stage, there is a low adherence noted after hospital discharge [1,15]. Indeed, the majority of patients adhere to the full programme while in hospital, with participation decreasing over time [16]. Furthermore, the proportion of patients who enrol in cardiac rehabilitation programmes as part of Phase II is relatively low, approximately 30% and during the course of the programme, the majority of patients withdraw [17,18]. Moreover, most patients who have undergone cardiac rehabilitation adopt healthier lifestyles during the course of their treatment. Nevertheless, upon returning to their everyday lives, patients gradually resume their pre-existing habits [1].

Numerous studies investigating the underlying causes of poor adherence to cardiac rehabilitation programmes have recognised that factors such as female gender, low education, and/or transportation issues are associated with an increased risk of withdrawal [17,18]. Moreover, in a study by Marzolini et al. (2008), it was observed that younger participants were more likely to demonstrate low adherence due to the demands of their work, while older participants were more committed to the programme, which is contrary to what was expected [17]. Indeed, this finding appears counterintuitive, as younger individuals are generally expected to exhibit greater adaptability and engagement in health-related activities. However, their professional and social obligations might restrict both their availability and motivation to consistently participate in rehabilitation programmes. In contrast, older participants have been reported to demonstrate higher adherence programmes, potentially due to a heightened awareness of health risks and fewer competing professional responsibilities. In contrast, a recent meta-analysis by Wang et al. (2023), which included 14 publications involving more than 114,000 patients [18], pointed out that the factors that were most likely to result in withdrawal from the programme were older age, a reduction in LVEF, a previous history of chronic heart disease, smoking habits, and the presence of hypertension and hyper-lipidaemia. The authors assumed that older age may be associated with lower participation in cardiac rehabilitation due to cognitive inability to comprehend the significance of this strategy for secondary prevention as well as a lack of educational qualifications and a low socio-economic status. It is interesting to note that, while both studies highlight age as a significant barrier, they reach contrasting conclusions regarding its impact. These divergent findings highlight the complexity of patient adherence to cardiac rehabilitation and suggest that tailored strategies are necessary to address the unique challenges faced by different age groups. For instance, younger patients might benefit from flexible scheduling or remote rehabilitation options that accommodate their work commitments, whereas older patients may require additional educational support and resources to enhance their compliance.

Furthermore, a factor that has been identified as a contributing element to the suboptimal adherence in cardiac rehabilitation programmes is the utilisation of antidepressant medication [17]. Yet, a Danish study confirmed that participants with low educational attainment, multiple comorbidities, transportation challenges, and solitary residence were more likely to abandon the programme; the participants with high anxiety and depression exhibited higher levels of adherence which is contrary to other studies [19].

One of the proposed strategies for maintaining high levels of adherence to cardiac rehabilitation programmes is the utilisation of telerehabilitation [1,20]. Given the significant barriers to participation in traditional cardiac rehabilitation programmes, such as transportation challenges and scheduling conflicts due to the job responsibilities, telerehabilitation offers a flexible and accessible alternative that can enhance patient adherence by allowing individuals to engage in rehabilitation from the comfort of their homes, thereby promoting sustained participation and improved health outcomes. Telerehabilitation is an approach that employs technology to provide remote rehabilitation services that encompass a range of modalities, including coaching, monitoring, and education. For instance, in the context of a remote exercise programme, it is recommended that supervised sessions are undertaken at a medical centre prior to commencement [20]. These sessions would facilitate the initial approach to rehabilitation and enable the patient to monitor their heart rate using devices that provide easily accessible measurements to the patient and the supervising professional [20]. Furthermore, an educational and psychological support programme should also begin with a physical interaction between the patient and the medical personnel. In both cases, subsequent sessions can be conducted remotely via online applications, telephone or through video consultation [20].

A number of meta-analyses, published approximately 10 years ago, have collated data from randomised controlled trials with the objective of evaluating the effects of telerehabilitation. According to these studies, telerehabilitation can be as effective as traditional rehabilitation [1,20]. Moreover, patients following telerehabilitation had an increase in adherence to physical activity, better functionality, and psychosocial well-being compared to those following centre-based cardiac rehabilitation [20]. However, most studies that evaluate telerehabilitation focus on the exercise component and adherence score. Consequently, further research is required to ascertain the impact of telerehabilitation on other aspects of cardiac rehabilitation [1].

Technological progress has given greater stimulus to remote assistance, which is now considered an opportunity to improve accessibility and adherence to cardiac rehabilitation. In addition, the experience of the global pandemic of COVID-19 has provided a unique opportunity to further explore the potential of alternative forms of training and assistance [21]. Indeed, telerehabilitation facilitates the participation of patients living at a distance from rehabilitation centres. Furthermore, the ability to follow programmes according to daily commitments is a significant advantage, which in turn reduces healthcare costs, and continuous monitoring provides updated data for tailoring programme adaptation [21].

It is of the utmost importance to emphasise that for the successful implementation of this novel tool, it is essential that medical practitioners are able to comprehend the nature of its functionality and dedicate the required time to study the system, as well as the opportunity to develop novel routines that facilitate optimal patient care [22].

**Figure 1:**
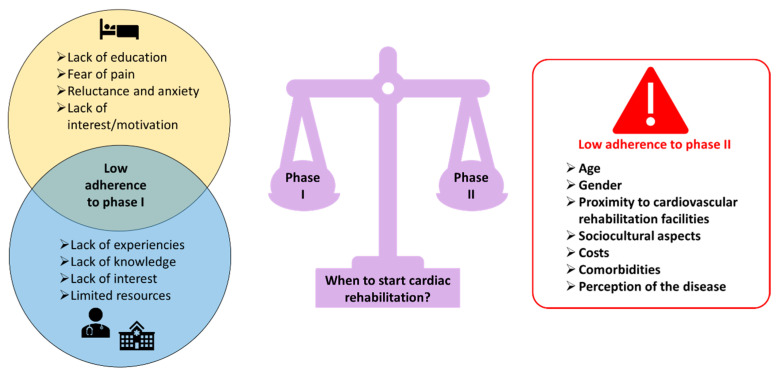
Reasons for low adherence to Phase I and Phase II.

Furthermore, it is worth mentioning here, as time passes following an ischemic event, patients frequently demonstrate inconsistency in other aspects of rehabilitation such as non-adherence to a healthy diet, cigarette consumption, alcohol abuse, and even prescribed medical therapies. Non-adherence to prescribed therapy has a negative impact on patient outcomes. The most effective means of reducing the risk of recurrent ischemic events hinges upon patients’ ability to maintain consistent adherence to prescribed treatment and lifestyle advice [23,24]. Unfortunately, approximately half of the patients after an acute MI tend to gradually decrease their adherence to prescribed drug therapy over time [23,24]. A number of factors contribute to the abandonment of therapy, including older age, female gender, educational level, financial support, healthcare access, disease acceptance, cognitive abilities, the complexity of prescribed therapy including polypharmacy and drug regimens, concerns about side effects and living alone [23,24]. Figure 1 provides a summary of the reasons for the low adherence to Phase I and Phase II.

Finally, it is equally important to address the limited resources available in many healthcare settings. Insufficient staffing, lack of specialised training, and inadequate infrastructure can significantly hinder the effective implementation of cardiac rehabilitation programmes. Healthcare professionals may not offer early mobilisation strategies due to a lack of expertise or time constraints, leading to missed opportunities for improving patient outcomes. Additionally, financial limitations may restrict access to necessary rehabilitation equipment and facilities. Therefore, a comprehensive approach that not only educates stakeholders, but also advocates for increased funding and resource allocation is essential to optimise the delivery of cardiac rehabilitation services and ensure that all patients receive the support they need for successful recovery.

## 4. Gender Differences in the Adherence to the Cardiac Rehabilitation Programmes

The evidence suggests that women diagnosed with CVD may experience inferior outcomes compared to men [17,18,25]. In general, women with CAD tend to be older and have a greater number of risk factors than men [17,18,25]. In the case of the presence of metabolic syndrome such as diabetes mellitus, dyslipidaemia, and/or obesity, the risk of CAD is increased further in women in comparison to men [25]. Nevertheless, it was observed that young women admitted for acute MI also exhibited a greater risk of in-hospital mortality compared with similarly aged counterparts [26,27]. Additionally, women exhibited a greater propensity for morbidity and mortality in the first year of recovery compared to men [26,27].

Yet, despite the well-established epidemiological evidence of gender differences in CVD prevalence and associated outcomes, the underlying processes that underpin these differences are not completely understood. However, it is possible to assert with certainty that gender plays a significant role in cardiovascular physiology and pathology, particularly in postmenopausal women, due to the reduction in oestrogen levels and the subsequent loss of its cardioprotective properties [28]. Further, it is pertinent to highlight that certain conditions such as adverse pregnancy outcomes including hypertensive disorders, gestational diabetes, preterm delivery, and/or pregnancy loss can have adverse effects on cardiovascular health in later life [29]. Also, women are more susceptible to conditions such as migraine, coronary spasms, and lupus erythematosus [25].

Finally, women frequently exhibit reduced physical activity, a lower socioeconomic status, and a heightened risk of adverse outcomes associated with smoking and diabetes compared to men [26]. Consequently, cardiovascular rehabilitation as secondary preventive care could effectively mitigate this burden; however, it is notable that cardiovascular rehabilitation in this specific population is largely underused. Indeed, a meta-analysis by Colella et al. (2015) comprising 19 observational studies revealed a significantly lower likelihood for women to be referred to outpatient cardiac rehabilitation compared to men [26]. More precisely, pooled analysis results indicated that the overall referral rate for men to a cardiovascular rehabilitation programme was approximately 1.5 times higher than for women [26]. In addition to the fact that women are less likely to be referred to cardiac rehabilitation programmes, it is worth mentioning that women are less likely than men to enrol [30] and adhere to such programmes [31].

**Figure 2:**
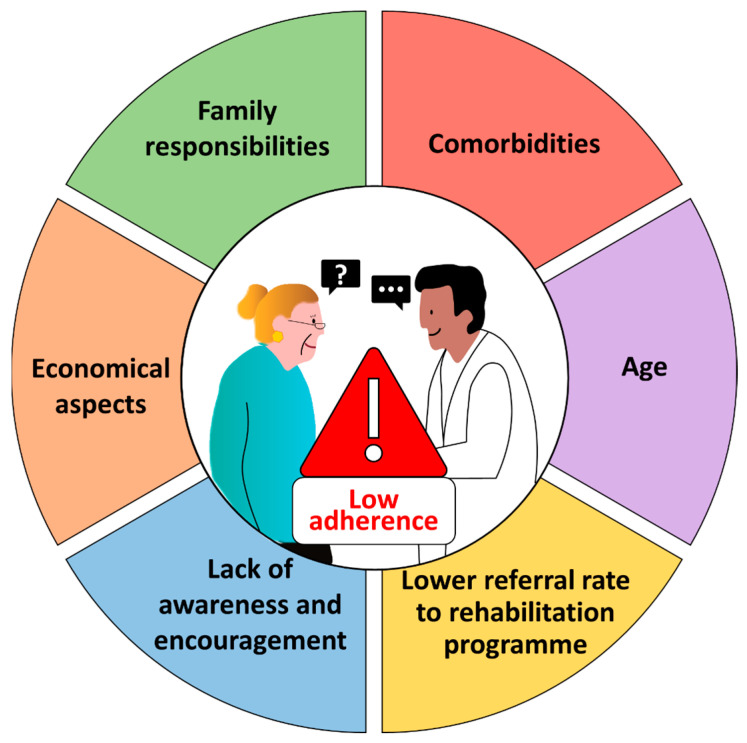
A summary of the factors contributing to the low adherence rates observed among women participating in cardiac rehabilitation programmes.

There has been a substantial number of qualitative studies conducted on the barriers to women engaging in cardiac rehabilitation, and the differences in these barriers between men and women (Figure 2) [32]. However, there has been a paucity of quantitative studies in this area. It is plausible to propose the most significant barriers to women engaging in cardiac rehabilitation are referral failure, lack of awareness, and the absence of encouragement and support from health providers [32]. These three factors may be the primary causes of this discrepancy, and crucial areas for intervention. First, increasing awareness among healthcare providers is essential to enhance women referrals to the programme. Second, targeted outreach efforts are necessary to improve programme participation among women. Lastly, implementing diverse support strategies, such as educational sessions and structured support systems, may further facilitate engagement and adherence. Furthermore, factors such as distance and transportation issues, the high burden of family responsibilities and obligations coupled with a lack of friends and family support, economic insecurity and healthcare insurance, and finally the perception that exercise is tiring or painful also contribute to the lower adherence of women to cardiac rehabilitation programmes [32,33]. Interestingly, a Canadian study by Marzolini et al. (2008) that evaluated gender differences in adherence to the 12-month cardiac rehabilitation programme, aside from the confirmed fact that women were more likely to withdraw from the programme, noted two interesting features. First, in both genders, significantly more participants younger than 55 were more prone to not completing the programme compared to their older counterparts [17]. Second, the multivariate regression analysis demonstrated that gender was not the primary factor influencing withdrawal from the programme. Rather, factors evaluated at baseline prior to the enrolment to the programme, including younger age, being unmarried, presence of obesity and diabetes mellitus, smoking habits, lower peak oxygen uptake, lack of previous coronary artery bypass graft surgery, use of antidepressant medication and lack of use of lipid-lowering or β-blockade medication, were found to be more significant [17]. After analysing the contributing parameters, the authors concluded that the profile of women was a significant factor in their decision to abandon the programme, rather than the sex itself. Nevertheless, it was noteworthy that in men, according to the results, lack of interest and work obligations were the primary obstacles to continuing with the cardiac rehabilitation programme, while in women, they included transportation issues and family responsibilities [17].

Finally, strategies to overcome these specific barriers, including motivational programmes, flexible working hours, and the use of alternative delivery models such as mobile devices, may prove beneficial in encouraging women to enrol in and persevere successfully through cardiac rehabilitation programmes [33]. It can be reasonably anticipated that the impact of such initiatives would be multi-faceted, encompassing enhanced well-being for participants, a reduction in recurrent events and morbidity and mortality rates, and a decline in healthcare expenditures. The utilisation of smartphone-based cardiac rehabilitation models that facilitate remote adherence and monitoring may be particularly advantageous for women [33].

In addition, it is of paramount importance to educate the general population on the benefits of regular exercise for overall health, with particular emphasis on CVD settings [34]. The use of media would be the optimal approach, as evidenced by the campaign and dissemination of information during the COVID-19 pandemic when healthcare professionals and media outlets advocated for the use of vitamin D due to its protective effects against respiratory infections [35]. Further studies are needed to assess the impact of this alternative delivery, given a notable lack of quantitative research exploring these issues comprehensively. Addressing this gap is essential for developing targeted interventions that can effectively promote adherence among female patients. Further studies are needed to assess the impact of this alternative delivery and ongoing support mechanisms, such as follow-up counselling and community engagement programmes, are essential in helping patients maintain healthier lifestyles post-rehabilitation.

## 5. Conclusions

In conclusion, the cardiac rehabilitation programme improves the cardiovascular health and reduces mobility and mortality risk in patients with CAD. After acute MI, a comprehensive cardiac rehabilitation programme should be started as early as possible while still in the hospital, as recommended by the European Society of Cardiology guidelines. Unfortunately, despite the proven benefits, cardiac rehabilitation programmes are underused for a variety of reasons, such as low referral rates by healthcare professionals, the inability of the centre to support such protocols. There is low patient interest in participating for multiple reasons, such as lack of awareness of the seriousness of the condition, perceived inability to understand both the condition and the benefits of cardiac rehabilitation, advanced age, female sex, transportation problems, financial aspects, education level, and/or health insurance. In addition, it has been observed that many patients adopt healthier lifestyles during rehabilitation, but as time passes after the ischemic event, patients tend to revert to previous bad habits that increase the risk of poor outcomes. Therefore, better strategies are needed to engage more patients in cardiac rehabilitation programmes and to ensure adherence. First, all guidelines should recommend Phase I of cardiac rehabilitation while patients are still in the hospital. This approach would increase the support for cardiac rehabilitation by healthcare professionals and increase the referral of patients to such protocols. Finally, there is also a need for an alternative form of support and cardiac rehabilitation such as telerehabilitation, which would overcome certain barriers such as transport, financial aspects and more flexible scheduling.
